# 

**Published:** 1917-10-20

**Authors:** 


### Manager's Notices.

**Now Ready, "The Hospital" Index to Vol. LXI., Oct. 1916 to April 1917. Price 2½d. per copy post free.**

**All letters on business matters must be addressed to The Manager, "The Hospital," 28 and 29 Southampton Street, London, W.C. 2.**

**Subscriptions**, which may commence at any time, are payable in advance. Cheques and Post-Office Orders should be crossed London County and Westminster Bank, Covent Garden Branch, and made payable to the Manager of The Hospital.

Rates of Subscription (payable in advance).

| | | |
|---|---|---|
| UNITED KINGDOM. | | |
| Three Months | (including Postage) ... ... ... ... | 2s. 0d. |
| Six Months | do. ... ... ... ... | 4s. 0d. |
| Twelve Months | do. ... ... ... ... | 6s. 6d. |
| FOREIGN AND COLONIAL. | | |
| Three Months | (including Postage) ... ... ... ... | 2s. 6d. |
| Six Months | do ... ... ... ... | 5s. 0d. |
| Twelve Months | do. ... ... ... ... | 8s. 6d. |

### The Book Market.

LIST OF NEW BOOKS RECEIVED FROM THE FOLLOWING PUBLISHERS:—

John Murray: "The Canteeners." By Agnes M. Dixon. 3s. 6d. net.

British Esperanto Association, 17 Hart Street, London, W.C. 1: "Esperanto and Why We Need It." By B. Long. 2d. net.

G. Bell and Sons: "British Boys: Their Training and Prospects." By Colonel King-Harman. 2s. net.

Simpkin, Marshall: "Gold Stripes." By Christie T. Young. 1s. net. "Observations of an Orderly." By "War Nurse." 2s. 6d. net.

John Wright and Sons, Ltd., Bristol; Simpkin, Marshall, London: "Blood Pictures." By Cecil Jones. 6s. 6d. net.

T. Fisher Unwin: "Me: A Book of Remembrance." 1s. net. "The Puppet." By Jane Harding. 6s.

The Modern Hospital Publishing Co., St. Louis, U.S.A.: "Modern Dietetics." By Lulu Graves.

G. P. Putnam's Sons: "Hygiene in Mexico." By Alberto J. Pani, C.E. 7s. 6d. net.

Baillière, Tindall and Cox, 8 Henrietta Street, W.C. 2: "The Jewish Child." By W. M. Feldman, M.B., B.D., London. 10s. 6d. net.

Cassell and Co.: "Hygiene and Public Health." By Sir Arthur Whitelegge, K.C.B., and Sir George Newman, M.D. 10s. 6d. net.

WE are asked to announce that the Chelsea Infirmary Nurses' League meeting is to be held at the Infirmary at 4 P.M. on Thursday, 25th instant.

### Matrons' and Sisters' Appointments.

Bath Union Infirmary.—Miss Charlotte Green has been appointed day sister. She was trained at St. Mary's Infirmary, Highgate Hill, N., where she was later staff nurse and ward sister.

Blackburn, Royal Infirmary.—Miss Edith E. Phillips has been appointed sister. She was trained at the Manchester Northern Hospital and at the Lincoln County Hospital, and has been night sister at the Derby Children's Hospital.

Children's Hospital, Derby.—Miss Louis Yates has been appointed night sister. She was trained at the Children's Hospital, Derby, and at the General Infirmary, Burton-on-Trent, where she has held the post of holiday sister.

Children's Hospital, Lower Sydenham.—Miss Beatrice Bertha Deane has been appointed sister. She was trained at the Kensington Infirmary, where she was later ward sister and night superintendent. She has also been staff nurse at Queen Mary's Hospital, Carshalton.

Red Cross Convalescent Hospital, Bryn Glas, Newport, Mon.—Sister H. Pryce has been appointed sister. She was trained at the West London Hospital.

Royal Salop Infirmary, Shrewsbury.—Miss Edith I. Walmsley has been appointed night superintendent. She was trained at the London Hospital, and has been matron's assistant and sister of the children's ward at the Royal West Sussex Hospital, Chichester.

Sandon Red Cross Hospital, Weston, Stafford.—Miss J. Vallance has been appointed night sister. She was trained at Glasgow Royal Infirmary, and has since been working at Greenock War Hospital.

**NOTE.—Particulars of Appointments for insertion in this column will be welcomed.**

### Passing Events and Topics.

### St. Thomas's Hospital Medical School.

Mr. R. H. O. B. Robinson, King's College, Cambridge, has been awarded the University Scholarship at St. Thomas's Hospital Medical School of £50.

### A Car for the Wounded.

Mr. and Mrs. W. H. Seager, of Cardiff, have presented the local Voluntary Aid Detachment with a car for the transport of wounded arriving at Cardiff and Whitchurch.

### Medical Officers' Salaries Increased.

The Huddersfield Board of Guardians at a recent meeting decided to increase the salaries of their town medical officers 25 to 50 per cent., and those of the country medical officers 20 to 25 per cent.

### A New Orphanage for Swansea.

Mr. Roger Beck, president of Swansea General Hospital, has set aside a gift of £10,000 towards the cost of erecting for Swansea a new orphanage. The Corporation proposes to place at the disposal of the trustees of the existing orphan home a suitable site, of 5¼ acres in extent, at a purely nominal rental.

### Middlesex Hospital Medical School.

The following have been awarded during the session 1916-1917.—*First Broderip Scholarship*, R. E. S. Webb; *Lyell Medal and Scholarship*, S. C. Shaw; *Freeman Scholarship*, S. C. Shaw; *Hetley Clinical Prize*, R. E. S. Webb; *Second Year's Exhibition*, O. S. Hillman; *First Entrance Scholarship*, H. B. Shaw; *Second Entrance Scholarship*, E. B. Dancy; *Third Entrance Scholarship*, J. Whitby.

### Gifts and Bequests.

Mr. Thomas Ridley, of Bury St. Edmunds, left £300 to the West Suffolk Hospital.

Miss Janet Macpherson, of Saville Row, Newcastle, dressmaker, left £100 to the Royal Victoria Infirmary, Newcastle.

Mr. Charles Otto Trechman, Ph.D., F.G.S., of Hudworth Tower, Castle Eden, Durham, left £100 to the Hartlepool Hospital.

Mr. John Ainsworth, of Ashton-upon-Mersey, left £100 each to Manchester Royal Infirmary and Manchester Royal Schools for the Deaf and Dumb.

Mr. H. R. Rhodes left £500 each to the British Home for Incurables, the Cancer Hospital, King Edward's Hospital Fund, the Royal Blind Pension Society, and the Royal Free Hospital.

Lieut. Andrew Richard Buxton, of the London Rifle Brigade, killed in action, a local director of Barclays Bank, left £500 for convalescent homes, to be selected, and £100 to the Royal Surgical Aid Society.

Stock to the value of £2,500 has been transferred to the Bradford Children's Hospital and the Royal Eye and Ear Hospital on account of the residue of the estate of Mr. John Holmes, divisible between the two hospitals.

Mr. Samuel Chambers, of Biggleswade, Beds, who left £8,405, bequeathed a few private legacies, and directed that the residue of his estate should be equally divided between the County Hospital, Bedford, and Dr. Barnardo's Homes.

Under the will of the late Mr. William Witton, of Wolverhampton, a large number of charities are benefited; they include the Wolverhampton Royal Orphanage, £1,200; the Hospital for Women, Wolverhampton, £600; and the Wolverhampton and Staffordshire General Hospital and the Eye Infirmary, £240 each.

Under the will of Mr. Walter Cliff, of York, a sum of £1,000 every six months, and a further £500 should the executors so direct, is to be accumulated for twenty-one years, and the *corpus* is to be applied for such charitable purposes, preferably for the benefit of the working classes, as the executors may select.

Mrs. M. A. Ingram, of Moseley, left a number of bequests to Birmingham charities, including £1,000 to the Birmingham and Midland Hospital for Children; £500 each to the Queen's Hospital, the General Hospital, the Royal Institute for the Blind, the Hospital for Women, the Ear Hospital, the Orthopædic Hospital, and the Eye Hospital.

Mrs. Ann Dudin Brown has left £10,000 for the endowment of scholarships at Westfield College, Hampstead; £1,000 each to the Royal Waterloo Hospital for Children and Women, Mount Vernon Hospital for Consumption, Brompton Hospital for Consumption, the National Hospital for the Paralysed and Epileptic, and the Cancer Hospital. Owing to the depreciation of securities it is possible that the legacies will not be paid in full.

Manchester Corporation has divided £2,000 amongst local hospitals in the following manner: Ancoats Hospital, £400; Chorlton-upon-Medlock Dispensary, £10; Christie Hospitals, £60; Ear Hospital, £60; Eye Hospital, £200; Hulme Dispensary, £50; Manchester Children's Hospital, £110; Manchester and Salford Sick Poor and Private Nursing Institution, £50; Northern Hospital, £100; Royal Infirmary, £550; St. Mary's Hospitals, £250; Hospital for Skin Diseases, £60; Dental Hospital, £50; Manchester Victoria Memorial Jewish Hospital, £50.

